# Metatranscriptomics of the human gut microbiome

**DOI:** 10.1186/gb-2011-12-s1-i15

**Published:** 2011-09-19

**Authors:** Thomas Sichertz Pontén

**Affiliations:** 1 Center for Biological Sequence Analysis, Department of Systems Biology, Technical University of Denmark, DK-2800 Kongens Lyngby, Denmark

## 

Our ‘other’ genome is the collective genetic information in all of the microorganisms that are living on and within us. Collectively known as the microbiome, these microbial cells outnumber human cells in the body by more than 10 to 1, and the genes carried by these organisms outnumber the genes in the human genome by more than 100 to 1.

How these organisms contribute to and affect human health is poorly understood, but the emerging field of metagenomics promises a more comprehensive and complete understanding of the human microbiome. In the European-funded Metagenomics of the Human Intestinal Tract (MetaHIT) project [1], we combined next-generation sequencing with high-density microarrays, generating metagenomic and metatranscriptomic data for more than 400 individuals.

The combined data reveal clusters of coexisting species with differences in pathway and gene function activity, suggesting that there is a division of labor between the bacterial species in the human gut microbiome.
